# Intensive therapy and remissions in rheumatoid arthritis: a systematic review

**DOI:** 10.1186/s12891-018-2302-5

**Published:** 2018-10-30

**Authors:** Catherine D. Hughes, David L. Scott, Fowzia Ibrahim, on behalf of TITRATE Programme Investigators, Heidi Lempp, Jackie Sturt, Louise Prothero, Isabel Neatrour, Rhiannon Baggott, Fowzia Ibrahim, Brian Tom, Allan Wailoo, James Galloway, Gabrielle Kingsley

**Affiliations:** Department of Rheumatology, King’s College London School of Medicine, Weston Education Centre, King’s College London, Cutcombe Road, London, SE5 9RJ UK

**Keywords:** Outcome, Early or established rheumatoid arthritis, Treatment response, Remission

## Abstract

**Background:**

We systematically reviewed the effectiveness of intensive treatment strategies in achieving remission in patients with both early and established Rheumatoid Arthritis (RA).

**Methods:**

A systematic literature review and meta-analysis evaluated trials and comparative studies reporting remission in RA patients treated intensively with disease modifying anti-rheumatic drugs (DMARDs), biologics and Janus Kinase (JAK) inhibitors. Analysis used RevMan 5.3 to report relative risks (RR) in random effects models with 95% confidence intervals (CI).

**Results:**

We identified 928 publications: 53 studies were included (48 superiority studies; 6 head-to-head trials). In the superiority studies 3013/11259 patients achieved remission with intensive treatment compared with 1211/8493 of controls. Analysis of the 53 comparisons showed a significant benefit for intensive treatment (RR 2.23; 95% CI 1.90, 2.61). Intensive treatment increased remissions in both early RA (23 comparisons; RR 1.56; 1.38, 1.76) and established RA (29 comparisons RR 4.21, 2.92, 6.07). All intensive strategies (combination DMARDs, biologics, JAK inhibitors) increased remissions. In the 6 head-to-head trials 317/787 patients achieved remission with biologics compared with 229/671 of patients receiving combination DMARD therapies and there was no difference between treatment strategies (RR 1.06; 0.93. 1.21). There were differences in the frequency of remissions between early and established RA. In early RA the frequency of remissions with active treatment was 49% compared with 34% in controls. In established RA the frequency of remissions with active treatment was 19% compared with 6% in controls.

**Conclusions:**

Intensive treatment with combination DMARDs, biologics or JAK inhibitors increases the frequency of remission compared to control non-intensive strategies. The benefits are seen in both early and established RA.

## Background

Remission has become a key treatment goal in rheumatoid arthritis (RA). Achieving remission with drug treatment is recommended in many clinical management guidelines [1–6]. It is also a central feature of the “treat-to-target” initiative [7, 8]. Patients who achieve remission have less disability and better quality of life than those with persisting inflammatory disease [9]. In early RA remission is particularly important due to the ‘window of opportunity’ during which early intensive treatment can halt or substantially reduce subsequent disease progression [10].

There are several definitions of remission in RA. The 2010 European League Against Rheumatism (EULAR) and American College of Rheumatology (ACR) criteria provided a framework for considering these different definitions [11]. A variety of composite measures are used to determine the presence of remission. These include the Disease Activity Score (DAS) and the Disease Activity Score for 28 joints (DAS28), the Simple Disease Activity Score (SDAI) and the Clinical Disease Activity Score (CDAI) [12–14]. DAS28 remission criteria have been used most frequently in trials of intensive treatments in RA, though there has been debate whether it is ideal [15].

Several systematic reviews have reported on treatment remissions in RA [16–20], patients likely to achieve remission [21, 22] and the strength of the rationale for treatment to target approaches in RA [23, 24]. The balance of evidence from these reviews is that intensive treatment increases remission. However, several uncertainties need to be resolved. Firstly, the relative merits of intensive treatment in early RA compared to established disease need to be considered. Secondly, it is important to know whether treatment with one type of therapy, such as biologics like tumour necrosis factor (TNF) inhibitors, will lead to more remissions than treatment with combinations of conventional disease modifying anti-rheumatic drugs (DMARDs) Finally it is important to know if one or other treatment strategy is preferable in early or established disease.

We have systematically reviewed RA clinical trials that report remissions. We evaluated both trials that compare an intensive treatment strategy with standard care and also head-to-head trials of different intensive treatment strategies. We analysed trials in early and established RA separately, taking the division between these groups as usually being 12 months since diagnosis.

## Methods

### Inclusion and exclusion criteria

The inclusion criteria were: randomized controlled trials or open label non-randomised comparative studies with at least one intensive treatment arm and one control arm; adult patients with RA; studies of at least 6 months duration; studies enrolling at least 50 patients; studies reporting remissions; studies using treatments in their licensed indication for RA. The intensive treatment arms used drugs considered more intensive than DMARD monotherapy. These included combination DMARDs (which could involve using short-term regular doses of steroids to control synovitis), TNF inhibitors, non-TNF biologics (tocilizumab, abatacept and rituximab), and Janus Kinase (JAK) inhibitors. We also noted whether studies used a treat-to-target approach with intensive treatments. Studies either compared one intensive treatment strategy against standard care or two different intensive treatment strategies (such as combination DMARDs and TNF inhibitors with DMARDs). Foreign language papers and published conference abstracts were excluded. Trials comparing similar types of treatment, such as two intensive DMARD regimens, were also excluded. The search identified publications from 1st January 2000 to 30th April 2017.

### Search strategy

A systematic literature search was carried out using EMBASE, OVID Medline as well as hand searching the systematic reviews relevant to this topic found in the Cochrane library database. The key word search terms used were ‘arthritis, rheumatoid’ (MeSH), ‘clinical trial’ [Publication Type] (MeSH), randomised controlled trial [Publication Type] (MeSH), open label (free text) and ‘remission’ (free text). These were searched separately and in combination. The EMBASE search terms included ‘arthritis, rheumatoid’ (MeSH) all subheadings and FOCUS function, clinical trial (MeSH) Explode function.

### Data collection

Two researchers (CH, DLS) independently assessed studies for eligibility and extracted data. This included year of publication, disease duration, number of treatment groups, study design, control and intensive treatment regimens, study size, remissions and study end-points. The numbers of patients achieving disease remission at the trial end-point was defined by Disease Activity Scores (DAS) < 1.6, DAS28 < 2.6 or equivalent criteria. The trials were classified as early (generally with disease durations < 1 year) or established (generally with disease durations > 1 year) reflecting the trial investigators assessments. When there were differences between assessors, they reviewed the reports together and came to a joint conclusion.

### Assessing Bias

A quality assessment was completed for each paper using the Cochrane Collaboration tool for assessing risk of bias [25]. The types of bias assessed were: random sequence generation, selection bias, performance bias, detection bias, attrition bias, reporting bias and other bias (such as pharmaceutical funding). The risk was defined as low or high. We also used funnel plots to assess publication bias and associated issues [26].

### Statistical analysis

Results were analysed using Review Manager 5.3 (Cochrane Collaboration, Oxford, UK). The random effects model based on DerSimonian and Laird’s method [27] was used to estimate the pooled effect sizes; this gives more equal weighting to studies of different precision in comparison with a simple inverse variance weighted approach, so accommodating between study heterogeneity. For all meta-analyses, we performed Cochrane’s chi-squared test to assess between study heterogeneity and quantified I^2^ statistics [25]. *P*-values < 0.05 were considered significant.

Some of the randomised controlled trials had more than two treatment arms: when there were two control groups the results were combined; when there were two or more intensive treatment groups only those reporting licensed dosage regimens were included.

## Results

### Study selection

**Fig. 1:**
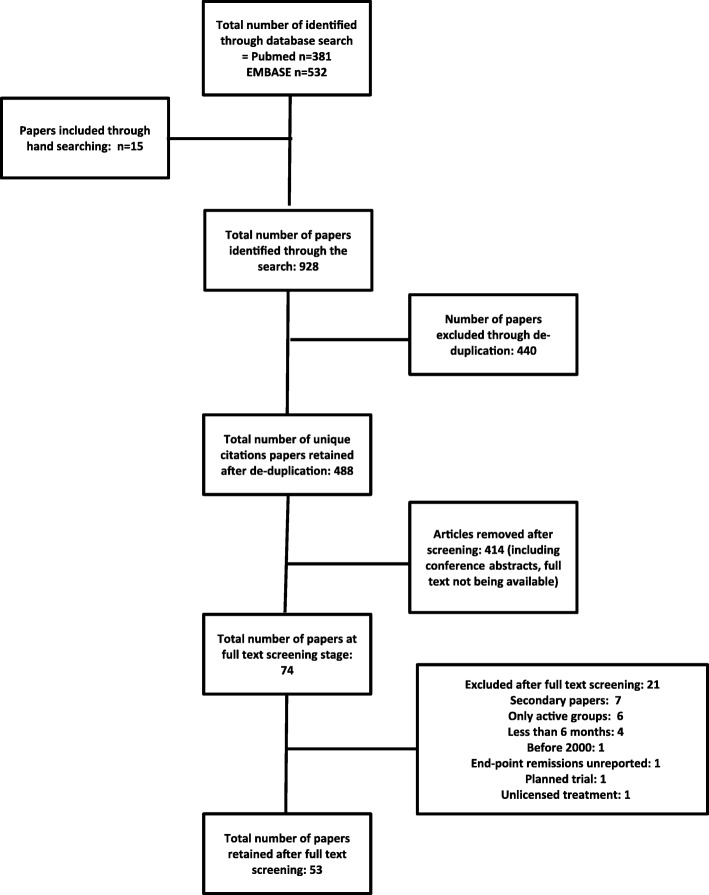
PRISMA Diagram Outlining Search Strategy

We identified 928 publications: 440 were duplicated studies and 414 were excluded after reviewing abstracts. Seventy four full text papers were reviewed in detail; 21 were excluded and 53 selected for inclusion (Fig. 1). These papers comprised 48 superiority trials, in which an intensive treatment strategy was compared with a less intensive strategy, and 6 head-to-head trials comparing combination DMARDs with biologic treatments. The BeST paper is included in both of these groups.

### Characteristics of included studies

Twenty two superiority trials evaluated patients reported as having early RA. Their maximum disease durations ranged from 3 months to 3 years. Mean or median disease durations, reported in 20 of these trials, ranged from 1 to 11 months (mean 6 months). Four of these trials studied patients with very early disease, less than 6 months from diagnosis. One trial had two different intensive treatment arms (combination DMARDs and biologics) which were both included. Six trials had two or three intensive treatment arms: in three trials biologic monotherapy treatment arms were omitted; in another three trials only licensed combination regimens were included.

Twenty six superiority trials evaluated patients with established RA. Six of these trials specified maximum disease durations (from 5 to 20 years). Mean or median disease durations, reported in all of these trials, ranged from 1 to 12 years (mean 8 years). One trial had two control groups (methotrexate or sulfasalazine monotherapy) and these were combined. Sixteen trials had two or more intensive treatment arms: three had two different licensed intensive treatments (biologics and JAK inhibitors) which were both included; in one trial the biologic monotherapy treatment arm was omitted; in a further 12 trials only licensed combination regimens were included.

Overall 19,752 RA patients were studied: 7300 in early RA and 12,452 with established RA (Table 1). There were 46 conventional RCTs, one was open label and one quasi-experimental. Twenty four trials had 2-arms, 17 had 3-arms and 7 had over three arms. The trials often reported outcomes at several different time-points, but their primary outcomes were reported at 6 months in 21 trials, at 12 months in 19 trials and at longer intervals in 8 trials (2 at 18 months, 5 at 24 months and 1 at 36 months).

**Table 1:** Details of Studies with Control Groups

First Author	Study	Year	Design	Groups	RA Duration	Quality Assessments			Months Follow-up	Treatments	
						Allocation	Blinding	Bias Analysis		Control	Intensive
Atsumi [28]	C-Opera	2016	RCT	2	Early	Low risk	Low risk	Low risk	12	MTX	Certolizumab/MTX
Bakker [29]	Camera II	2012	RCT	2	Early	Low risk	Low risk	Low risk	24	MTX	Prednisolone/MTX
Bijlsma [30]	U-Act-Early	2016	RCT	3	Early	Low risk	Low risk	Low risk	24	MTX	Tocilizumab/MTX
Breedveld [31]	Premier	2005	RCT	3	Early	Low risk	Low risk	Low risk	24	MTX	Adalimumab/MTX
Burmester [32]	Function	2015	RCT	4	Early	Unclear	Unclear	Low risk	12	MTX	Tocilizumab/MTX
Capell [33]	Mascot	2007	RCT	3	Est’lishd	Low risk	Low risk	Low risk	12	MTX or SZP	MTZ/SZP
Cohen [34]	Reflex	2006	RCT	2	Est’lishd	Low risk	Low risk	Low risk	6	MTX	Rituximab/MTX
Detert [35]	Hit Hard	2012	RCT	2	Early	Low risk	Low risk	Low risk	6	MTX	Adalimumab/MTX
Dougadas [36]	Act-Ray	2013	RCT	2	Est’lishd	Low risk	Low risk	Low risk	6	Tocilizumab	Tocilizumab/MTX
Emery [37]	Avert	2014	RCT	3	Early	Low risk	Low risk	Low risk	12	MTX	Abatacept/MTX
Emery [38]	Comet	2008	RCT	2	Early	Low risk	Low risk	Low risk	12	MTX	Etanercept/MTX
Emery [39]	Go Before	2009	RCT	4	Est’lishd	Low risk	Low risk	Low risk	6	MTX	Golimumab/MTX
Emery [40]	Radiate	2008	RCT	3	Est’lishd	Low risk	Low risk	Low risk	6	MTX	Tocilizumab/MTX
Emery [41]	Serene	2010	RCT	3	Est’lishd	Low risk	Low risk	Low risk	12	MTX	Rituximab/MTX
Emery [42]	C-Early	2017	RCT	2	Early	Low risk	Low risk	Low risk	12	MTX	Certolizumab/MTX
Genovese [43]	RA Beacon	2016	RCT	3	Est’lishd	Low risk	Low risk	Low risk	6	DMARD	Baracitinib/DMARDs
Genovese [44]	Toward	2008	RCT	2	Est’lishd	Low risk	Low risk	Low risk	6	DMARD	Tocilizumab/DMARD
Goekoop Ruitermann [45]	BeSt	2005	RCT	4	Early	Low risk	Low risk	Low risk	12	DMARDs	Infliximab/DMARDs or Combination DMARDs
Grigor [46]	Ticora	2004	RCT	2	Est’lishd	Low risk	Low risk	Low risk	18	Usual Care	Combination DMARDs
Hetland [47]	Cimestra	2006	RCT	2	Early^a^	Unclear	Low risk	Low risk	12	MTX	MTX/Ciclosporin
Horslev Petersen [48]	Opera	2014	RCT	2	Early^a^	Low risk	Low risk	Low risk	12	MTX	Adalimumab/MTX
Kavanaugh [49]	Optima	2013	RCT	2	Est’lishd	Low risk	Low risk	Low risk	6	MTX	Adalimumab/MTX
Kivitz [50]	Brevacta	2014	RCT	2	Est’lishd	Low risk	Low risk	Low risk	6	DMARD	Tocilizumab/DMARD
Klareskog [51]	Tempo	2004	RCT	3	Est’lishd	Low risk	Low risk	Low risk	6	MTX	Etanercept/MTX
Kremer [52]	–	2005	RCT	3	Est’lishd	Low risk	Low risk	Low risk	12	MTX	Abatacept/MTX
Kremer [53]	Lithe	2011	RCT	3	Est’lishd	Low risk	Low risk	Low risk	24	MTX	Tocilizumab/MTX
Kremer [54]	–	2012	RCT	7	Est’lishd	Low risk	unclear	Low risk	6	MTX	Tofacitinib/MTX
Kremer [55]	–	2013	RCT	4	Est’lishd	Low risk	Low risk	Low risk	6	DMARD	Tofacitinib/DMARD
Nam [56]	Empire	2014	RCT	2	Early^a^	Low risk	Low risk	Low risk	12	MTX	Etanercept/MTX
Nam [57]	Idea	2014	RCT	2	Early	Low risk	Low risk	Low risk	18	MTX	MTX/infliximab
Schiff [58]	Attest	2007	RCT	3	Est’lishd	Low risk	Low risk	Low risk	12	MTX	Abatacept/MTX or Infliximab/MTX
Schipper [59]	–	2012	Quasi-Exp	2	Early	High risk	High risk	Indeterminate	12	Usual care	Tight control^b^
Smolen [60]	Certain	2014	RCT	2	Est’lishd	Low risk	Low risk	Low risk	12	DMARD	Certolizumab/DMARD
Smolen [61]	Go After	2009	RCT	3	Est’lishd	Low risk	Low risk	Low risk	6	DMARD	Golimumab/DMARD
Smolen [62]	Option	2008	RCT	3	Est’lishd	Low risk	Low risk	Low risk	6	MTX	Tocilizumab/MTX
Smolen [63]	Rapid2	2008	RCT	4	Est’lishd	Low risk	Low risk	Low risk	6	MTX	Certolizumab/MTX
Soubrier [64]	Guepard	2009	RCT	2	Early^a^	Low risk	High risk	Unclear	12	MTX	Adalimumab/MTX
St. Clair [65]	–	2004	RCT	3	Early	Low risk	Low risk	Low risk	12	MTX	Infliximab/MTX
Symmons [66]	Brosg	2006	RCT	2	Est’lishd	High risk	Low risk	Low risk	36	Symptomic	Combination DMARDs
Tak [67]	Image	2010	RCT	3	Early	Low risk	Low risk	Low risk	12	MTX	Rituximab/MTX
Takeuchi [68]	Hopeful-1	2014	RCT	2	Early	Low risk	Low risk	Low risk	6	MTX	Adalimumab/MTX
Taylor [69]	RA Beam	2017	RCT	3	Est’lishd	Low risk	Low risk	Low risk	6	MTX	Baracitinib/MTX or Adalimumab/MTX
van der Heijde [70]	Oral Scan	2013	RCT	3	Est’lishd	Low risk	Low risk	Low risk	6	MTX	MTX/Tofacitinib
Van Ejik [71]	Stream	2012	RCT	2	Early	Uncertain	Low risk	Low risk	24	Usual care	Intensive treatment
van Vollenhoven [72]	Oral Standard	2012	RCT	4	Est’lishd	Low risk	Low risk	Low risk	6	MTX	Tofacitinib/MTX or Adalimumab/MTX
Verstappen [73]	Camera	2007	Open label	2	Early	High risk	High risk	Indeterminate	24	Usual care	Combination DMARDs
Weinblatt [74]	Go Further	2013	RCT	2	Est’lishd	Low risk	Low risk	Low risk	6	MTX	Golimumab/MTX
Westhovens [75]	–	2009	RCT	2	Early	Low risk	Low risk	Low risk	12	MTX	Abatacept/MTX

a. These trials enrolled patients with disease durations no more than 6 months. b. In Schipper et al. study by 12 months 16% controls had combination DMARDs and 6% had TNF inhibitors; with intensive treatment 30% had combination DMARDs and 12% TNF inhibitors. The trial was classified as comparing combination DMARDs

Abbreviations: *RCT* Randomised controlled trial, *Est’lishd* Established, *MTX* Methotrexate, *SZP* Sulfasalazine, *DMARD* Disease modifying anti-rheumatic drugs

DAS28 remissions (DAS28 < 2.6) were reported in 38/48 superiority trials and 4/6 head-to-head trials. DAS remissions (DAS < 1.6) were reported in 5/48 superiority trials and 2/6 head-to-head trials. Five superiority trials reported other remissions (using SDAI in 3 and unique study-specific criteria in 2). In addition, 12 superiority trials reported some or all of the new EULAR/ACR remission criteria.

Treat-to-target strategies were included within 8/48 superiority trials and 3/6 head-to-head trials, though there were substantial differences in how these strategies were delivered.

### Remission in superiority trials

Overall in the 48 trials 3013/11,259 patients achieved remission with intensive treatment compared with 1211/8493 patients receiving non-intensive therapy (Table 2). Analysis of the 53 comparisons in these trials using the random effects relative risk model showed there was a highly significant benefit for intensive treatment (RR 2.23; 95% CI 1.90, 2.61). There was marked heterogeneity between studies; I2 was 84%.

**Table 2:** Effectiveness In Superiority Trials Assessed By Random Risk Ratio and Heterogeneity

	Treatments	Trials	Comparisons	Random Risk Ratio (95% CI)	Heterogeneity
All	All	48	52	2.23 (1.90, 2.61)	I^2^ = 84%
	DAS28 Remissions	38	40	2.26 (1.89, 2.71)	I^2^ = 85%
	Other Remission Criteria	10	12	2.13 (1.53, 2.98)	I^2^ = 81%
	6 Month Duration	21	24	3.78 (2.60, 5.51)	I^2^ = 86%
	12 Month Duration	19	20	1.73 (1.44, 2.09)	I^2^ = 82%
	18–36 Month Duration	8	8	1.84 (1.39, 2.42)	I^2^ = 79%
	Used TTT Strategy	8	9	1.62 (1.30, 2.03)	I^2^ = 75%
Early	All^a^	22	23	1.56 (1.38, 1.76)	I^2^ = 74%
	TNF Inhibitors	13	13	1.44 (1.26, 1.66)	I^2^ = 62%
	Other Biologics	5	5	2.00 (1.53, 2.63)	I^2^ = 79%
	Combination DMARDS^b^	5	5	1.46 (1.11, 1.93)	I^2^ = 73%
	Used TTT Strategy	6	7	1.51 (1.22, 1.88)	I^2^ = 72%
Established	All	26	29	4.21 (2.92, 6.07)	I^2^ = 86%
	TNF Inhibitors	10	10	3.59 (2.14, 6.03)	I^2^ = 70%
	Other Biologics	10	10	6.81 (2.62, 17.7)	I^2^ = 95%
	Combination DMARDS	3	3	2.41 (1.14, 5.10)	I^2^ = 67%
	JAK Inhibitors	6	6	3.39 (2.14, 5.36)	I^2^ = 0%
	Used TTT Strategy	2	2	2.39 (0.90, 6.32)	I^2^ = 83%

^a^The 4 very early trials which enrolled patients with disease durations no more than 6 months involved 4 comparisons with a random risk ratio (95% CI) of 1.47 (1.03, 2.10) and I^2^ 72%

^b^Excluding the Schipper et al. study in which some patients in both groups had DMARD monotherapy, DMARD combination therapy and TNF inhibitors leaves 4 trials with 4 comparisons with a random risk ratio (95% CI) of 1.38 (1.01, 1.88) and I^2^ 71%

Abbreviations: *DAS28* Disease Activity Score for 28 joints, *TNF* Tumour necrosis factor, *DMARDs* Disease modifying anti-rheumatic drugs, *JAK* Janus kinase, *TTT* Treat To Target

In the 38 trials (40 comparisons) reporting DAS28 remissions the random risk ratio was 2.26 (95% CI 1.89, 2.71); in the 10 trials (12 comparisons) reporting other remission criteria the random risk ratio was 2.13 (95% CI 1.53, 2.98). The random risk ratios showed significant effects with trials of 6 months, 12 months and longer durations. Although the random ratio was somewhat higher in trials of 6 months duration, 17/21 trials (20/24 comparisons) were in established RA and in these the random risk ratio was 4.82 (95% CI 2.85, 8.13); in the 4 trials (4 comparisons) lasting 6 months in early RA the random risk ratio was 1.94 (95% CI 1.21, 3.11). In the 8 trials (9 comparisons) involving TTT strategies as part of intensive treatment the random risk ratio was 1.62 (95% CI 1.30, 2.03).

In the 22 trials in early RA with intensive treatments trials with 1756/3993 patients achieved remission with intensive treatment compared with 903/3307 patients receiving monotherapy. One trial evaluated two intensive treatment regimens and there were consequently 23 comparisons; 13 evaluated TNF inhibitors, 5 evaluated other biologics and 5 evaluated combination DMARDs. Analysis of the 23 comparisons in these trials showed a significant overall benefit for intensive treatment (RR 1.56; 95% CI 1.38, 1.76). There was marked heterogeneity in these studies; I^2^ was 74% (Table 2). A funnel plot showed a symmetrical pattern in these trials (result not shown). Four trials enrolled patients with disease durations no more than 6 months and these showed a similar benefit for intensive treatment (RR 1.47; 95%CI 1.03, 2.10) Comparison of the different intensive treatment regimens in early RA patients showed similar impacts of different intensive treatments; these ranged from a random risk ratio of 1.43 with TNF inhibitors to 2.00 with other biologics. TTT strategies also increased remissions with a random risk ratio of 1.51.

In the 26 established RA trials 1257/7266 patients achieved remission with intensive treatment compared with 308/5186 patients receiving monotherapy. Three trials evaluated two intensive treatment regimens and consequently there were 29 comparisons: 10 evaluated TNF inhibitors, 10 evaluated other biologics, 3 evaluated combination DMARDs and 6 evaluated JAK inhibitors. Analysis of these 29 comparisons trials showed a significant overall benefit for intensive treatment (RR 4.21; 95% CI 2.92, 6.07). There was marked heterogeneity in these studies; I^2^ was 86% (Table 2). A funnel plot showed an asymmetrical pattern in these trials (result not shown). Comparison of the different intensive treatment regimens in established RA patients showed some differences in the magnitude of effects; random risk ratios ranged from 2.41 with combination DMARDs to 6.81 with other biologics (tocilizumab, adalimumab and rituximab); however, as the confidence intervals overlapped there was no evidence these differences were significant. Only two trials used TTT strategies and although these increase remissions the 95% confidence intervals showed the finding may not have been significant (random risk ratio 2.39; 95% CI 0.90, 6.32).

Using a fixed effects model gave similar findings. In all trials the risk ratio was 2.06 (95%CI 1.94, 2.18), in early RA trials it was 1.64 (95% CI 1.54, 1.74) and in established RA the risk ratio was 3.32 (95% CI 2.94, 3.74). Interestingly the fixed model indicated TTT strategies in established RA in two trials may have been significant (risk ratio 2.19, 95% CI 1.50, 3.19.

### Remission in head to head trials

Overall in the 6 trials 317/787 patients achieved remission with TNF inhibitors compared with 229/671 of patients receiving combination DMARD therapies. Analysis of these 6 trials using the random effects relative risk model (Table 3) showed there was a no different between treatment strategies (RR 1.06; 95% CI 0.93. 1.21). There was little heterogeneity between studies; I^2^ was 21%. Comparing 4 early RA and 2 established RA trials separately also showed no evidence of a significant difference between groups (Table 3). However, comparisons of the first 6 months results in the two established RA trials showed more remissions with TNF inhibitors using the random effects relative risk model (RR 1.74, 95% CI 1.14, 2.64). The fixed effects model gave similar findings (RR 1.90; 95% CI 1.17, 3.10).

**Table 3:** Effectiveness In Head To Head Trials Comparing Biologic with Combination DMARD Strategies Assessed By Random Risk Ratio and Heterogeneity

	Trials	Random Risk Ratio (95% CI)	Heterogeneity
All	6	1.06 (0.93, 1.21)	I^2^ = 21%
Early	4	1.05 (0.88, 1.24)	I^2^ = 40%
Established	2	1.21 (0.88, 1.68)	I^2^ = 0%
Established First 6 Months	2	1.74 (1.14, 2.64)	I^2^ = 0%

### Frequency of remissions

**Fig. 2:**
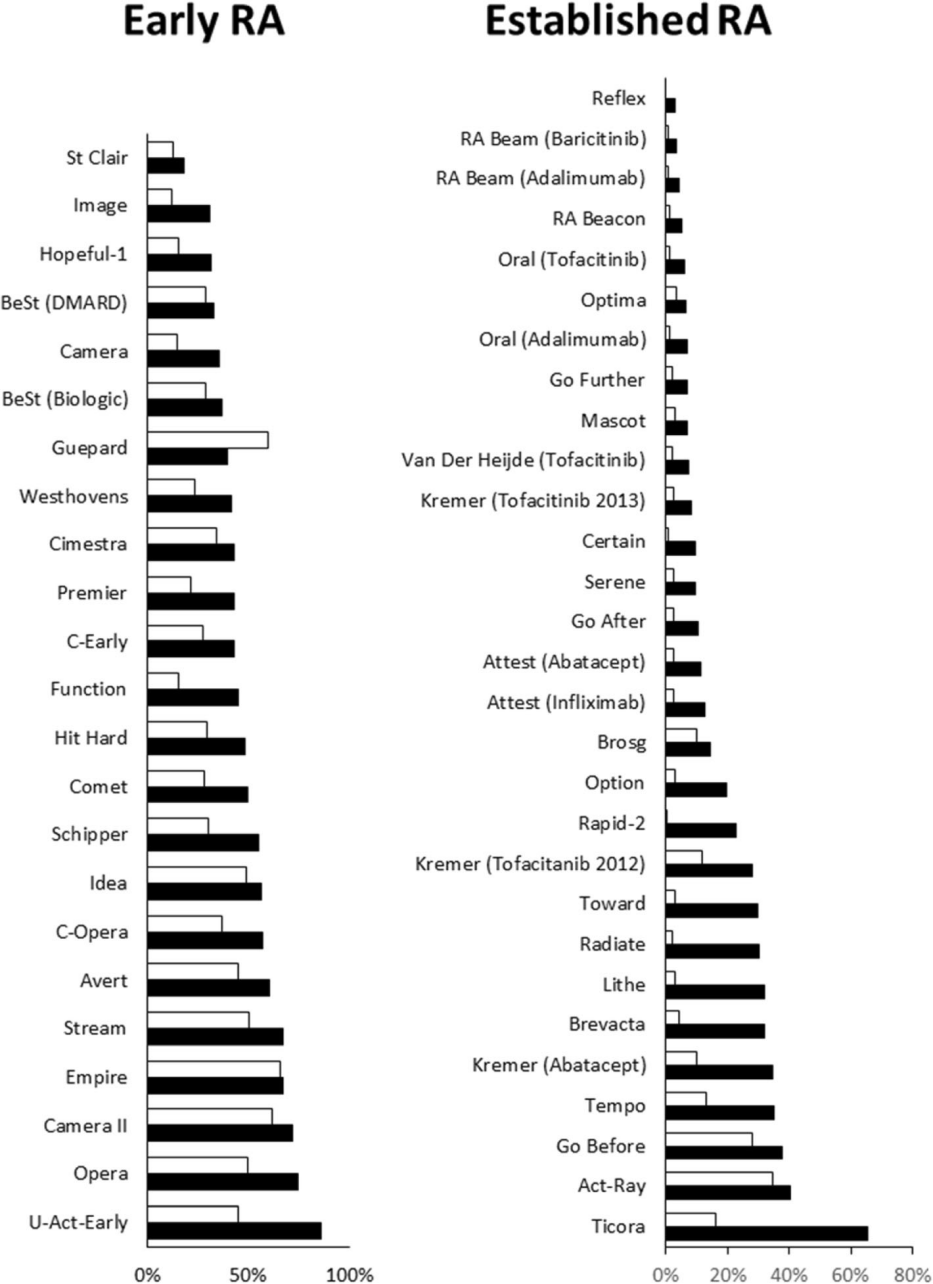
Remissions in control and active groups shown as percent patients in each group in early and established RA

There were marked differences in the frequency of remissions in active and control groups in both early and established RA (Fig. 2). In early RA the average frequency of remissions with active treatment was 49%: in 10 early RA trials 50% or more active patients achieved remissions; the highest rate was 86% in the U-Act-Early (tocilizumab) trial and the lowest rate was 18% in the St Clair (Infliximab) trial. In early RA controls the average frequency of remission was 34%: in four trials 50% or more controls achieved remissions; and the lowest rate in controls was 18% in the Image (rituximab) trial. The average difference in remission rates between active and control group in early RA trials was 15%.

In established RA the average frequency of remissions with active treatment was 19%: in only one trial did 50% or more active patients achieved remission (65% in the Ticora trial of combination DMARDs); in 14 trials 10% or less active patients achieved remission and, in the Reflex, (rituximab) and RA Beam (baricitinib and adalimumab) trials only 3% of patients achieved remissions. In established RA controls the average frequency of remission was 6%: in 22 trials less than 5% of controls achieved remissions; and in the Reflex (rituximab) trial no control patient achieved an end-point remission. The average difference in remission rates between active and control group in early RA trials was 13%.

### Quality and risk of Bias

Quality assessment, using the Cochrane Collaboration tool for assessing risk of bias, showed overall quality was high with low risks of bias (Table 1).

## Discussion

TNF Inhibitors, other biologics and combination DMARDS were all effective in increasing remission in early and established RA. Treat to target strategies, which usually involved intensive DMARDs, were also effective. JAK inhibitors were similarly effective in established RA; there was no data about their impact in early disease. Although other biologics achieved numerically higher risk ratios in both early and established RA the overlapping confidence intervals gave no support to the view that these differences are clinically significant. The benefits of different types of intensive treatment were therefore broadly similar. Trials of varying durations, from 6 months to more than 12 months, all showed intensive treatments increased remissions. There was no evidence that patients with very early RA of no more than 6 months disease duration benefited more from intensive treatments. We excluded trials with durations of less than 6 months to ensure we did not disadvantage the assessment of intensive treatment strategies using slower acting DMARDs. The head-to-head trials supported the similarities between treatments with combination DMARD strategies and TNF inhibitor strategies, which achieved similar end-point remission rates. There was however, some evidence that TNF inhibitors increased the early remission rates, in keeping with their relatively rapid onset of action compared to conventional DMARD combinations.

TNF Inhibitors, other biologics and combination DMARDS were all effective in increasing remission in early and established RA. There was no evidence that patients with very early RA of no more than 6 months disease duration benefited more from intensive treatments. JAK inhibitors were similarly effective in established RA; there was no data about their impact in early disease. Although other biologics achieved numerically higher risk ratios in both early and established RA the overlapping confidence intervals gave no support to the view that these differences are clinically significant. The head-to-head trials supported the similarities between treatments with combination DMARD strategies and TNF inhibitor strategies, which achieved similar end-point remission rates. There was however, some evidence that TNF inhibitors increased the early remission rates, in keeping with their relatively rapid onset of action compared to conventional DMARD combinations.

The overall quality of the studies was relatively high. However, there was evidence of marked heterogeneity in their findings with most comparisons having high I^2^ values. This heterogeneity meant that in some intensive treatment arms in early RA over 70% patients achieved remission while in other intensive treatment arms in established RA under 10% patients achieved remission. These differences are likely to reflect patient selection more than treatment efficacy, with very early RA patients having no previous DMARDs are highly likely to achieve remission with intensive treatment while established RA patients who have failed multiple prior treatments are unlikely to do so.

The most likely explanation for the asymmetrical funnel plot in trials in established RA relates to specifically including studies using treatments in their licensed indication which were published between 2000 and 2017. A consequence is that potential intensive treatments which were evaluated in RA patients but were not found to be effective, were not included. Firstly, small initial studies with new drugs which would have shown negative results for remissions were not included as the treatments were never licensed for RA. An example is the spleen tyrosine kinase inhibitors [81]. Secondly, some TNF inhibitors were not effective in RA and were therefore not licensed; an example is Lenercept, which failed to show sustained benefit in clinical trials [82]. Finally, combinations with DMARDs were tried in the 1980’s, before remission was measured or reported; these trials were mainly negative [83]; subsequent trials of intensive DMARD combinations reporting remission which were published after 2000 studied treatments which were known to be effective in combination. These factors mean the funnel plot of remissions in established RA would not include small trials with negative findings because of the selection criteria used. As this report focuses on the benefits of different intensive treatment strategies using licensed treatments given at their approved dosages we do not think an asymmetric funnel plot changes our conclusions.

Our systematic review has a number of limitations. Firstly, studies not reporting remission data were excluded, though they often show clinically important improvements with intensive treatment. Secondly, studies reported remissions differently; for example, DAS and DAS28 remissions are similar but not identical. Thirdly, studies were of variable duration and comparing remission rates over 6 and 12 months or more is not ideal; however, variations in treatments and patient selection meant there was no evidence for one particular time point being best. Fourthly, studies differed in the way they handled non-responders, with some trials stopping treatment if patients had not responded within 3 months or so and applying non-responder imputations. This approach may alter the remission rates in the non-intensive treatment by making it appear smaller than it might have been if treatment was continued. Fifthly, as mentioned previously, studies enrolled different patient groups in whom the likelihood of achieving remissions was very different. Sixthly, the intensive combination DMARD regimens used in the trials have been combined together, even though they represent a wide range of different strategies, not all of which appeared highly effective. In one study by Schipper et al. [58] some patients in active and control groups had monotherapy and others had biologics, so the trial is not just a comparison of one treatment strategy; however, excluding it made no difference to the conclusions. Finally, there is debate about the benefits of combining the results of different trials in a meta-analysis, considering their potential degrees of clinical heterogeneity. As we have also undertaken extensive sub-group analyses we consider the approach we have taken is justified in this particular clinical context.

Our results have several implications for clinical practice. Firstly, they show that intensive treatment strategies lead to more remissions than conventional care in both early and established RA. This finding is generally supportive of the treat-to-target approach currently recommended [7, 8], although we have not attempted to dissociate the impact of giving intensive treatment from the impact of the target. Secondly, they show that initial treatment with conventional DMARD combinations has similar effectiveness to early biologics. This finding is supportive of the current recommendations about biologic treatments from the National Institute for Health and Care Excellence (NICE), which recommend trying combination DMARDs before biologic treatment [84]. Thirdly, they question whether remission is necessarily the ideal target for treatment in established RA, as it is only achieved by a minority of patients in most trials of intensive treatment. There may be greater value of aiming for low disease activity states, in which case these need to be measured in future trials. The EULAR good response criteria can be used to assess the frequency of achieving low disease activity states measures using DAS28. Current guidance on treat to target includes aiming for low disease activity in some patients.

One issue this review cannot address is treatment sequencing. Some experts believe most early RA patients should receive methotrexate monotherapy initially for a few months and only have intensive treatments if they fail to respond. Other experts recommend early intensive treatment followed by treatment tapering. It is possible to find individual trials within our systematic review, which support both options, but there is no systematic evidence to support or refute either approach. One final pair of inter-related uncertainties is the optimal time to assess remission and the most suitable assessment to evaluate its presence. Combining superiority and head-to-head trials (Tables 1 and 4) shows 23 (43%) lasted 12 months, 20 (38%) lasted 6 months and 10 (19%) lasted over 12 months, with the longest (BROSG trial evaluating combination DMARDs) lasting 3 years. This finding suggests trials of 12 months or longer seem preferable. Although most trials reported DAS28 remissions, this represents an historical target and there is now greater emphasis on stricter remission criteria.

**Table 4:** Details Of Head-To-Head Studies

First Author	Study	Year	Design	Groups	RA Duration	Quality Assessments			Months Follow-up	Treatments	
						Allocation	Blinding	Bias Analysis		Non-Biologic	Biologic
Goekoop Ruitermann [45]	BeSt	2005	RCT	4	Early	Low risk	Low risk	Low risk	12	Combination DMARDs	Infliximab/DMARDs
Heimans [76]	Improved	2014	RCT	2	Early	Low risk	High risk	Unclear	12	Triple DMARDs	Adalimumab/MTX
Leirisalo-Repo [77]	Neo-Fin RA Co	2013	RCT	2	Early	Low risk	Low risk	Low risk	24	Triple DMARDs	Infliximab/Triple DMARDs
O’Dell [78]	Racat	2013	RCT	2	Est’lishd	Low risk	Low risk	Low risk	12	Triple DMARDs	Etanercept/MTX
Scott [79]	Tacit	2015	RCT	2	Est’lishd	Low risk	High risk	Indeterminate	12	Combination DMARDs	TNF inhibitors/DMARDs
Moreland [80]	Tear	2012	RCT	4	Early	Low risk	Low risk	Low risk	24	Triple DMARDs	Etanercept/MTX

Abbreviations: *RCT* Randomised controlled trial, *Est’lishd* Established, *MTX* Methotrexate, *DMARD* Disease modifying anti-rheumatic drugs, *TNF* Tumour necrosis factor

## Conclusions

Intensive treatment with combination DMARDs, biologics or JAK inhibitors increases the frequency of remission compared to control non-intensive strategies. The benefits are seen in both early and established RA. The relative merits of different remission criteria in trials is a complex question but changing criteria has the disadvantage of making it difficult to compare trials with newer criteria and those using more historic methods.

## Abbreviations

ACR: American College of Rheumatology
BROSG: British rheumatoid outcome study group
CDAI: Clinical disease activity score
CI: Confidence intervals
DAS: Disease activity score
DAS28: Disease activity score for 28 joints
DMARDs: Disease modifying anti rheumatic drugs
EULAR: European league against rheumatism
JAK inhibitors: Janus kinase inhibitors
NICE: National Institute for Health and Care Excellence
RA: Rheumatoid arthritis
RR: Relative risks
SDAI: Simple diseasee activity score
TNF inhibitors: Tumour necrosis factor inhibitors
TTT: Treat to target
